# Supplementary material for: Patterns of Intron Gain and Loss in Fungi
Source: PLoS Biol. 2004 Nov 30;2(12):e422. doi: 10.1371/journal.pbio.0020422 (PMC532390; doi:10.1371/journal.pbio.0020422)
Supplement: Table S1 — Also available at http://genes.mit.edu/NielsenEtAl/. (4.3 MB ZIP). [file pbio.0020422.st001.zip › NielsenEtAl/html/89.html]

AN3758.1.NCU03296.1.MG04544.1.FG05598.1


```
 CLUSTAL W (1.82) Multiple Sequence Alignments - Introns Inserted


Sequence 1: NCU03296.1	341 aa
Sequence 2: MG04544.1	345 aa
Sequence 3: FG05598.1	335 aa
Sequence 4: AN3758.1	336 aa
Alignment Length: 376 aa
Number Identitical Residues: 104 aa
Alignment Score (without introns) 5937


MG04544.1 	MAAAGLSRAAAPMLRQSISRCRPLQLFKQ--QRRWAEVHDIRFLTTTRPRQNIAEKYREK
NCU03296.1	MASL---RTAG-MLRHLAHPCSHVRTLRASNQRRWAQVHDVRFLATTQQPRSVIEKYRQK
FG05598.1 	MASL---RIPA-LRRLMAAPSRATRAVNQ---RRWAQVHDIRFLATTQPSQAVVEKYREK
AN3758.1  	MASIR-------SFTLQCLPWSRSSLIRS-QQRRWAQVHDVRFLATHHDPNQVLDRYRAK
          	**:                       ..  ..****:***:***:* :  . : ::** *

MG04544.1 	LDRKAKS~EGLTGIDELKQAYAERIKEQRKAA-AAIAPPGLDK--ILADEPAARATQTVE
NCU03296.1	LENKAKE~EGLPDIDALKQAYKDKIERLRKETDAATKLPGAPAPATPTAADAAELDATTT
FG05598.1 	LSQKAQQ~EGLESIEQLKAAYSAKIDAERRKG-AVEFPPGTIP--QAPETPVIQPNADNP
AN3758.1  	LDQRAKQ2EGFDSVEALKKAHQEKIDKLRREASTVLTP----------EPPAPSKKPHSP
          	*..:*:. **: .:: ** *:  :*.  *:  .:.                .        

MG04544.1 	SGANTAGTPTEQSSNSYSKPETTPAKDSSGSQVKPLSAIVDLPKVRQLPVPELTEIWRLL
NCU03296.1	TTTEAAKPSSNATPETPKKPVSSGSGSGSGSGIKPLSEILDMTKARDLPIKELSAIWRLR
FG05598.1 	PGQDQPNKDTRDPPASPSKPPPTGADK---QAIKTLNDIIDLPKVRELPEKELTAIWRLR
AN3758.1  	PPPPKPQSAAQSQVAAAAKSVSS-------SGIKPLGSYLDVEKVRALPPKEIEAIWRLR
          	.    .   :.    :  *. .:       . :*.*.  :*: *.* **  *:  **** 

MG04544.1 	HTTDPKSLCAVVPAPTYKAMEALARQRPQFVLPVPHPEQ----------------GAEMH
NCU03296.1	YATNPHSLCAVIPSETYSAMDTLARSRPQFVLPVPHPEQ----------------GAEIH
FG05598.1 	HASSEQNLCAVIPTSTYKAMEDAARNAPQFVLPVPHPAQ----------------GAEIH
AN3758.1  	HASNSNSICAVIPLETYQRIASAARQNPQFILPLPRSASESQSSEAETTATGDKGGADIH
          	:::. :.:***:*  **. :   **. ***:**:*:. ..:.::.:.::::....**::*

MG04544.1 	FLQWTFD--------AATGTSTVLFTQLAEYKNRGEFAQPHTTVTHHPDLADDKGVVLMQ
NCU03296.1	FLQWTWD--------AATATSTVLFTQLAEYKNRGEFAQPHTTVTHYMDFSVDKGVVLMQ
FG05598.1 	FLQWTFD--------AQSKTSTVLFTQLAEYKNRGEFAQPHTTITHHLDLANDRGLVLMQ
AN3758.1  	FLQWAFHPPASAPSAVNTHTSTVIFTPLAAYKLHGAYAQPHTTITHHLDLADDKGLVLMH
          	****::...:::.::. : ****:** ** ** :* :******:**: *:: *:*:***:

MG04544.1 	GSMVDNRGVKIADAQWLVMCLQRFYGGWDPALSAGGSRLQSEERAKARR-ELVEWFGTGN
NCU03296.1	GQVMEDRGVKPEDAQWLLMCLQRFYGGWD---GTGGE--QGQQRANERR-NLLDWFASGD
FG05598.1 	GHVLPDRGVTPENAKWLLMSLQRFYGGWEG--ETAELSGERKDRAEERK-KLLSWFAAGD
AN3758.1  	GQVMPDSGVSTAEATWLVSCVQRFY--------------DFGGQASGRKGELLRMFTQGD
          	* :: : **.  :* **: .:****              :   :*. *:.:*:  *  *:

MG04544.1 	-ENFSIEKLLAEAERLG
NCU03296.1	-QRFSVEKLMEEAERMG
FG05598.1 	-GRFSVDKLLEEAERMG
AN3758.1  	VQNFRIEELMAEAEKL-
          	  .* :::*: ***::
```
